# Germs and Acids in Decay of the Teeth

**Published:** 1883-07

**Authors:** J. S. Cassidy


### Germs and Acids in Decay of the Teeth.

BY PROF. J. S. CASSIDY, D.D.S.

(Read before the Kentucky State Dental Society.)

So much thought has been expressed of late in favor of and against accepting the so-called "germ" theory of diseases in general, and of dental caries in particular, that I would hesitate to say anything on the subject were it not for an honest desire to accept both sides of the question and try to reconcile, in my own mind at least, the differences in the relationship between the germ and the acid, whether sought for in the human mouth, or in other conditions, favorable to spontaneous passage of highly organized compounds toward what we call simpler forms of matter.

The influence of minute organisms in causing putrefaction or decay has been recognized as a fact almost since "the morning stars sang together." Of late years the microscope has demonstrated the existence of many distinct and indistinct species of the genus bacteria, and, perhaps, each in its own sphere and circumstance really does promote the disease with which it is credited. There still remains, however, the open question for science to answer: "In what way is their influence accomplished?"

All modifications of force effect chemical changes, easily or otherwise, according to laws and conditions, known or unknown, of mutual affinity between the reacting substances; and "vital" force, whether animal or vegetable, is the most persistent in its selective action, overcoming adverse physical conditions of matter, in order to assimulate that by which the principle of life may be sustained. With bacteria, organized structure, or the debris of such, is required for food, much as with animals of more pretension; they cannot construct protein from the mineral kingdom; hence inorganic material neither putrifies nor ferments. Neither would organized matter, when excluded from germinal influence, suffer the spontaneous decomposition implied by the processes of putrefaction and fermentation—terms, by the way, having the same general chemical significance, except the smell.

It is well known that the average human mouth is peculiarly favorable to harbor all the requisites for active changes. Eight different kinds of bacteria have been detected there, any one of which at the normal temperature can induce molecular disturbance in the semi-liquid, highly organized residue of food mixture and mucus. It is a most convenient and luxurious dish for them, as indeed are all other similar mixtures and more or less devitalized tissues generally. But in what way is the degenerative metamorphosis induced? Is it by the removal of certain constituents from the mass in order to nourish and multiply the bacteria, or is it that they bring with them the essence of catalytic influence, which by mere contact, changes the already predisposed complex fluid, or living tissue, into their own proper food, thereby, in the case of fluids, extraneous or otherwise, reducing the residual or most easily affected organic compounds into simpler forms, and with living tissue provoking inflamation and perhaps specific disease? The latter view seems to be in consonance with the general idea of germ influence, premising, however, that the substance or part affected thus, must in all cases be in such perfect physical and chemical condition as to furnish the bacteria with their natural and sufficient nourishment.

Now in all fairness, in the light of experience and common sence, is a tooth, alive or dead, ever in such condition? We answer most emphatically, no. Nor do many of the germ theorists claim such to be the case, a few, at least, admitting that the bacteria must first find little holes in the enamel into which they, the bacteria, crawl, while a large majority, according to my reading, admit that the enamel must first be softened by an acid in order to permit the little animals to feed on the sweet gelatinous portion of the interior, and by such feeding merely comes the disease known as dental caries in all its varieties. Is not this, so far, a fair statement?

On the other side, let us agree that it is the chemical side; the fact has always been well known that an organism of some kind was a necessity to fermentation. But fermentation is neither more nor less than chemical change, and, though the substances be whatever they may, if the process be allowed to continue uninteruptedly to the end, it will be found to consist, first, of due preparation for the reception of germs, then rapidly passing through several stages, each stage producing its own peculiar compounds, until finally the acid stage is reached, at which point the process, per se, is at an end. Let a given mixture be prepared as food for bacteria, and we can foretell nearly all the several compounds which will ensue, down to the typical acid of the special process. But with such mixtures of animal and vegetable matters as must of necessity exist, much or little, in the omnivorous human mouth, as soon as decomposition begins, the results are modified, not alone by the vicissitudes peculiar to the part, but also by the oft-recurring changes in the composition of the heterogeneous mixture itself, so that no prediction can be made from day to day as to the probable result; the process is beyond our control. There is, however, one important fact to remember, that an acid is the usual ending of every process of fermentation; and there is no truth in chemistry of which I am more fully convinced than that an acid in its *nascent* state is the beginning of dental caries, and indeed the only solvent throughout the entire course of the disease. A cavity in a tooth, so begun, is a very favorable place for continued fermentation, by the extraneous matters it receives and can retain, and by the consequent irritation and inflamation of its own organic structure. Of course living organisms are present also, for, as truly remarked, "putrefaction is a concomitant not of death, but of life."

The claim that analysis has proven the normal quantity of calcium salts still in a carious cavity is absurd, as every dentist surely knows; but even if comparatively large quantities of calcium phosphate were occasionally found in the devitalized part, which I do not doubt, its presence can be easily explained by remembering that hydrochloric acid does not decompose calcium phosphate; it simply dissolves it, the salt chemically unchanged being susceptible to complete precipitation from solution by the ammonia which is formed in the localized putrefaction.

For the sake of brevity I have purposely avoided mention of the names of authorities, and have not entered into details as to any of the probable reactions in the mouth, save the one just alluded to, nor of the acids most likely to be developed there. The object for which I wrote thus briefly being merely to see how a firm believer in the insidious universal influence of germs can consistently vote aye on the acid doctrine of dental caries.

